# Multiple Functional Motifs Are Required for the Tumor Suppressor Activity of a Constitutively-Active ErbB4 Mutant

**Published:** 2013-08-22

**Authors:** Richard M. Gallo, Ianthe N. Bryant, Christopher P. Mill, Steven Kaverman, David J. Riese

**Affiliations:** 1 Department of Medicinal Chemistry and Molecular Pharmacology, Purdue University College of Pharmacy & Purdue University Center for Cancer Research, West Lafayette, IN 47907, USA; 2 Department of Microbiology and Immunology, Indiana University School of Medicine, Indianapolis, IN 46206 USA; 3 Department of Pharmacal Sciences, Auburn University Harrison School of Pharmacy, Auburn, AL 36849-5501 USA

**Keywords:** ErbB4/HER4, Signal Transduction, Tumor Suppressor, Protein Trafficking

## Abstract

ErbB4 (HER4) is a member of the ErbB family of receptor tyrosine kinases, which includes the Epidermal Growth Factor Receptor (EGFR/ErbB1), ErbB2 (HER2/Neu), and ErbB3 (HER3). Mounting evidence indicates that ErbB4, unlike EGFR or ErbB2, functions as a tumor suppressor in many human malignancies. Previous analyses of the constitutively-dimerized and –active ErbB4 Q646C mutant indicate that ErbB4 kinase activity and phosphorylation of ErbB4 Tyr1056 are both required for the tumor suppressor activity of this mutant in human breast, prostate, and pancreatic cancer cell lines. However, the cytoplasmic region of ErbB4 possesses additional putative functional motifs, and the contributions of these functional motifs to ErbB4 tumor suppressor activity have been largely underexplored. Here we demonstrate that ErbB4 BH3 and LXXLL motifs, which are thought to mediate interactions with Bcl family proteins and steroid hormone receptors, respectively, are required for the tumor suppressor activity of the ErbB4 Q646C mutant. Furthermore, abrogation of the site of ErbB4 cleavage by gamma-secretase also disrupts the tumor suppressor activity of the ErbB4 Q646C mutant. This last result suggests that ErbB4 cleavage and subcellular trafficking of the ErbB4 cytoplasmic domain may be required for the tumor suppressor activity of the ErbB4 Q646C mutant. Indeed, here we demonstrate that mutants that disrupt ErbB4 kinase activity, ErbB4 phosphorylation at Tyr1056, or ErbB4 cleavage by gamma-secretase also disrupt ErbB4 trafficking away from the plasma membrane and to the cytoplasm. This supports a model for ErbB4 function in which ErbB4 tumor suppressor activity is dependent on ErbB4 trafficking away from the plasma membrane and to the cytoplasm, mitochondria, and/or the nucleus.

## Introduction

**Figure 1:**
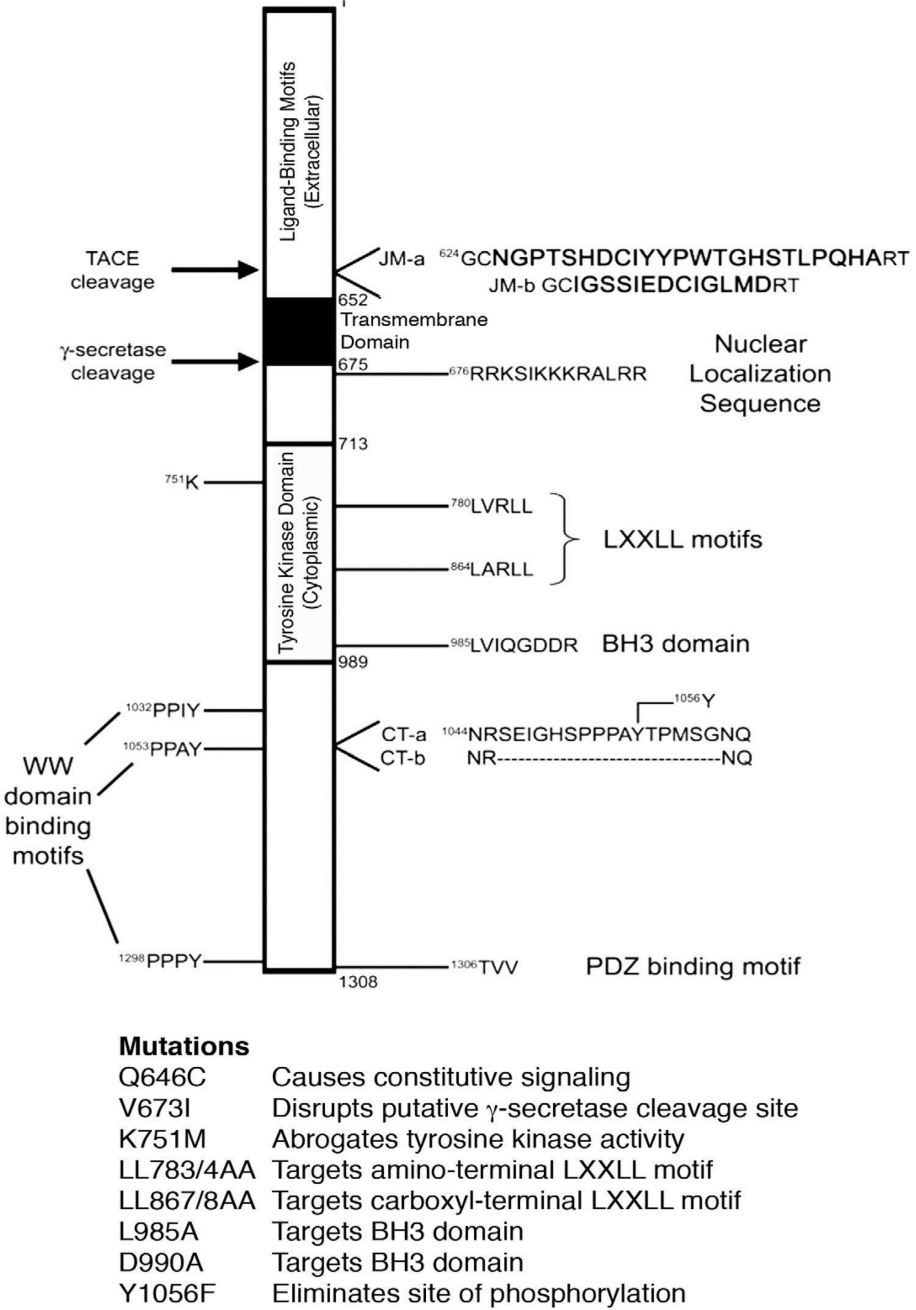
ErbB4 Possesses Multiple Functional Motifs and Mutations Have Been Engineered to Target These Motifs The organization of ErbB4 is as indicated in this schematic. The extracellular ligand-binding motifs reside in the amino-terminal region upstream of amino acid residue 651. The single-pass transmembrane domain consists of amino acid residues 652–675. The cytoplasmic tyrosine kinase domain consists of amino acid residues 713–989. The majority of cytoplasmic sites of tyrosine phosphorylation reside in amino acid residues 990–1308, most notably Tyr1056. Additional putative functional motifs include a TACE cleavage site, a gamma-secretase cleavage site, two LXXLL (steroid hormone receptor binding) motifs, a BH3 domain, three WW domain binding motifs, and a PDZ domain binding motif. Mutations that disrupt these motifs are noted. Finally, note the two locations of alternative transcriptional splicing, resulting in a total of four different splicing isoforms.

ErbB4 (HER4) is a member of the ErbB family of receptor tyrosine kinases, a family that also includes the Epidermal Growth Factor Receptor (EGFR/HER1), ErbB2 (HER2/Neu), and ErbB3 (HER3). Like other members of the ErbB family, ErbB4 possesses extracellular ligand-binding motifs, a hydrophobic transmembrane domain, and a cytoplasmic tyrosine kinase domain (Figure 1). Ligands for ErbB4 are members of the Epidermal Growth Factor (EGF) family of peptide growth factors. Binding of one of these ligands to ErbB4 causes receptor dimerization, phosphorylation of multiple cytoplasmic tyrosine residues of ErbB4, ErbB4 binding to effectors that possess SH2 or PTB binding motifs, and activation of multiple downstream signaling pathways [1–4].

There are two sites of alternative splicing of the ErbB4 transcript, one in the juxtamembrane (JM) region and one in the cytoplasmic tail (CT) region, giving rise to four distinct ErbB4 isoforms [5, 6]. Relative to the canonical JM-a/CT-a isoform (aka JM-a/Cyt1), the JM-b isoforms possess an alternative short sequence in the extracellular juxtamembrane region of the protein (Figure 1). The CT-b isoforms lack a short sequence in the cytoplasmic region of the protein, distal to the tyrosine kinase domain.

Aside from the ligand-binding, transmembrane, and tyrosine kinase domains, ErbB4 possesses a number of other motifs that may be critical for ErbB4 function (Figure 1). These include sites for cleavage by Tumor Necrosis Factor Alpha-Converting Enzyme (TACE) and gamma-secretase [5, 7–9], a nuclear localization sequence [10], LXXLL motifs (which may enable interactions with nuclear hormone receptors) [11–13], a BH3 domain [14] (which enables binding to Bcl family proteins), motifs for binding to WW domains [15, 16], and a motif for binding to PDZ domains [17, 18]. The JM-b isoforms do not contain the canonical site for cleavage by TACE; the CT-bisoforms lack a putative site of phosphorylation (Tyr1056) and a motif for binding to WW domains. EGFR and ErbB2 are potent oncoproteins whose aberrant signaling is associated with a number of human malignancies [2–4, 19–23]. In contrast, there is much evidence from clinical studies and laboratory investigations indicating that ErbB4 possesses tumor suppressor ivities [14, 24–39]. We have previously generated the ErbB4 Q646C mutant, which displays ligand-independent dimerization and tyrosine phosphorylation [40, 41].

This mutant inhibits clonogenic proliferation of several human breast, prostate, and pancreatic tumor cell lines [40, 42, 43]. This tumor suppressor activity requires a functional ErbB4 kinase domain as well as Tyr1056 [42, 44].

Multiple reports indicate that a recombinant ErbB4 cytoplasmic domain functions as a tumor suppressor, suggesting that cleavage of the native ErbB4 protein by Tumor Necrosis Factor alpha Converting Enzyme (TACE) and/or gamma-secretase and release of the ErbB4 intracellular domain (4ICD or s80 fragment) from the plasma membrane are required for ErbB4 tumor suppressor activity [35, 45–50]. However, heretofore we have not established whether the other ErbB4 functional motifs found in the cytoplasmic region of ErbB4 are required for the tumor suppressor activity of the ErbB4 Q646C mutant. Thus, here we use ErbB4 mutants to demonstrate that additional ErbB4 motifs are required for the ErbB4 Q646C mutant to inhibit clonogenic proliferation of breast and prostate tumor cell lines. We also present data that suggest some of the ErbB4 mutants defective for tumor suppressor activity also exhibit aberrant subcellular localization. Thus, we postulate that the tumor suppressor activity of the constitutively-active ErbB4 mutant may be regulated by subcellular trafficking.

## Materials and Methods

### Cell lines and cell culture

Mouse C127 fibroblasts and the Ψ2 and PA317 recombinant retrovirus packaging cell lines are generous gifts of Dr. Daniel DiMaio (Yale University, New Haven, Connecticut, USA). These cells were cultured essentially as described [41, 43, 51]. The following cell lines were obtained from the American Type Culture Collection (ATCC): PC-3 and DU-145 human prostate tumor cell lines, the MCF7 human mammary tumor cell line and the MCF10A human mammary epithelial cell line. These cell lines were cultured in accordance with ATCC recommendations. Cell culture media and supplements were obtained from GE Biosciences (formerly GIBCO/BRL/Life Technologies). Fetal bovine serum and G418 were obtained from Gemini Bioproducts. Plasticware and Giemsa stain were obtained from Fisher Scientific.

### ErbB4 mutants

Unless otherwise noted, all ErbB4 constructs used in this paper were generated in the context of the recombinant retrovirus expression vector pLXSN-ErbB4 [52], which encodes the canonical 1308-amino acid, human ErbB4 JM-a CT-a isoform. The constitutively-dimerized and –active Q646C ErbB4 mutant has been previously described [40, 41], as have the kinase-deficient K751M ErbB4 mutant and the Y1056F ErbB4 phosphorylation site mutant [42, 44]. Additional ErbB4 mutants were generated by standard site-directed mutagenesis and subcloning techniques (Figure 1). The sequence of each mutagenic primer is available upon request.

The ErbB4 V673I mutation, in which the Val673 codon is changed to an isoleucine codon, disrupts ErbB4 cleavage by gamma-secretase [35]. The ErbB4 LL783/4AA mutation, in which the Leu783 and Leu784 codons are both simultaneously changed to an alanine codon, is predicted to disrupt ErbB4 binding to nuclear hormone receptors [11–13]. The ErbB4 LL867/8AA mutation, in which the Leu867 and Leu868 codons are both simultaneously changed to an alanine codon, is also predicted to disrupt ErbB4 binding to steroid hormone receptors. The ErbB4 L985A and D990A mutations, in which the Leu985 and Asp990 codons are each changed to an alanine, are predicted to disrupt ErbB4 binding to Bcl family proteins [53]. ErbB4 expression and tyrosine phosphorylation was detected by immunoprecipitation and immunoblotting as previously described [40, 41, 54].

### ErbB4-EGFP fusion constructs

The ErbB4 Enhanced Green Fluorescent Protein (EGFP) fusion construct, in which the EGFP sequence is added to the carboxyl terminus of the ErbB4, has been previously described [10] and is the generous gift of Dr. Frank Jones (Tulane University, New Orleans, Louisiana, USA). To generate the ErbB4 Q646C EGFP construct, the DraIII-HpaI fragment of this construct, which encodes the carboxyl-terminal portion of ErbB4 and the entire EGFP sequence, was ligated to the DraIII –BamHI fragment of pLXSN-ErbB4-Q646C [41], which encodes the recombinant retroviral sequences and the aminoterminal portion of ErbB4, including the Q646C mutation. We used standard site-directed mutagenesis to add the sequence that encodes Thr-Val-Val to the 3’ end of the sequence that encodes the EGFP tag.

### Inhibition of clonogenic proliferation by ErbB4

This assay was performed essentially as described [40, 42–44]. Briefly, all ErbB4 constructs were expressed from the recombinant retroviral expression vector pLXSN, which carries the neomycin resistance gene [55] and is a generous gift of Dr. Daniel DiMaio (Yale University). A high-titer amphotropic retroviral stock was generated from each construct using standard techniques, the Ψ^2^ ecotropic retrovirus packaging cell line, and the PA317 amphotropic retrovirus packaging cell line [56, 57]. The DU-145, PC-3, MCF7, and MCF10A cell lines were infected with these viruses and stably infected cells were selected using G418. By comparing the number of drug-resistant colonies that arise from infection with the tester viruses to the number of drug-resistant colonies that arise from infection with the control viruses (typically the pLXSN vector control virus or the virus encoding the kinase-deficient [K751M] ErbB4 Q646C mutant), we assessed the effects of the ErbB4 mutants on clonogenic proliferation of these human prostate and breast cell lines.

Our analysis of what we term clonogenic proliferation is formally a combination of plating efficiency and the ability of the seeded cells to proliferate into a visible colony of drug-resistant cells. We have previously demonstrated that the failure of MCF7 cells that have been infected with the pLXSN-ErbB4-Q646C retrovirus to form colonies of drug-resistant cells does not appear to be due to a diminished plating efficiency, but rather is a consequence of growth arrest [42]. Consequently, our analysis of what we term clonogenic proliferation is likely to primarily be an analysis of clonogenic proliferation and not of plating efficiency. Analyses of these data were performed as described elsewhere [40]. Briefly, we normalized for differences in virus titer by infecting C127 fibroblasts in parallel; this permitted us to calculate the efficiency of each virus in causing clonogenic proliferation in the tester cell lines (“clonogenic efficiency”) by dividing the titer of a given viral stock in the tester cell line by the titer of that same viral stock in the C127 control cell line. (In Tables 1–4 we report the mean “clonogenic efficiency” values calculated from at least three independent experiments.) Thus, those tester viruses that exhibit lower “clonogenic efficiency” than the control viruses (LXSN or ErbB4 in Tables 1 and 2; ErbB4 Q646C K751M in Table 3; ErbB4 Q646C K751M and ErbB4 Q646C EGFP in Table 4) are judged to inhibit clonogenic proliferation.

To make these judgments more obvious, we have also calculated “inhibition of clonogenic proliferation” for each tester virus by dividing the “clonogenic efficiency” of that tester virus by the “clonogenic efficiency” of the corresponding control virus and subtracting that result from 100%. This value was calculated for each trial of at least three independent experiments. The mean and the standard error of these values are reported. The larger the mean value, the more the mutant behaves as an inhibitor of clonogenic proliferation.

### ErbB4 subcellular localization

Various pLXSN-ErbB4-EGFP constructs (200 ng, uncut plasmid) were transiently transfected into the PC-3 human prostate tumor cell line using TurboFectin 8.0 (Origene). Cells were incubated for 48 hours, after which they were stained with Hoescht 33342 (Sigma - for DNA) and MitoTracker Red CMXRos (Molecular Probes - for mitochondria). Cells were imaged using a Radiance 2100 MP Rainbow (BioRad) laser scanning confocal unit attached to a TE2000 inverted microscope with a 60× oil-immersion objective lens (Nikon). The MitoTracker Red (red images) was imaged following excitation at 543 nm using a green HeNe laser; the fluorescence emission greater than 560 nm was collected. EGFP (green images) was imaged following excitation with the 488 nm line of the 4-line Argon laser; the fluorescence emission was collected using a 500LP/550SP filter combination. Hoescht33342 (blue images) was imaged following excitation at 350 nm using a Mai Tai laser (Spectra Physics); the emission between 420 and 480 nm was collected. We photographed multiple randomly-selected GFP-positive cells per transfected plasmid per experiment. Photomicrographs shown are representative of results from at least three independent experiments.

## Results

### The K751M, V673I, LL783/4AA, and L985A mutations profoundly disrupt the tumor suppressor activity of the constitutively- active ErbB4 Q646C mutant

We have previously constructed a constitutively-dimerized and -active form of the ErbB4 receptor tyrosine kinase by substituting a cysteine residue for Gln646 of the ErbB4 extracellular juxtamembrane region (ErbB4 Q646C mutant - Figure 1). The ErbB4 Q646C mutant inhibits clonogenic proliferation of several human cell lines, including the DU-145 and PC-3 human prostate tumor cell lines [43, 44], the SKBR3 and MCF7 human breast tumor cell lines, the MCF10A human breast epithelial cell line [42], and the CaPan-1, HPAC, MIA-PaCa2, and PANC-1 human pancreatic tumor cell lines [40]. This tumor suppressor activity displayed by the ErbB4 Q646C mutant requires ErbB4 tyrosine kinase activity and the carboxyl-terminal, cytoplasmic Tyr1056 residue [42, 44]. However, it is not known whether other putative ErbB4 functional motifs are required for the tumor suppressor activity of the ErbB4 Q646C mutant.

**Figure 2:**
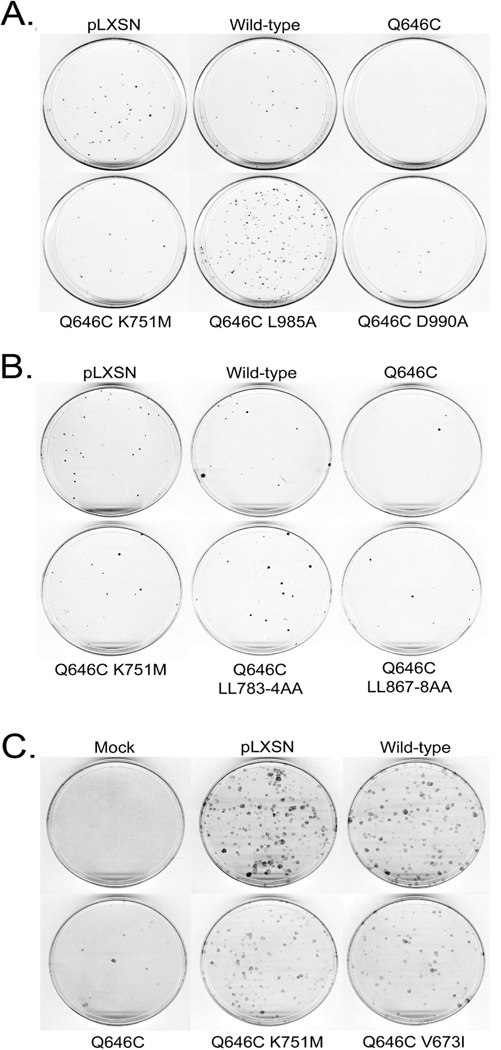
K751M, V673I, LL783/4AA, and L985A mutations profoundly disrupt the tumor suppressor activity of the constitutively-activeErbB4 Q646C construct in MCF7 and MCF10A cell lines (a,b) MCF7 cells were infected with recombinant retroviruses based on the pLXSN vector as indicated. Infected cells were selected using G418 and colonies of infected, drug-resistant cells were stained using Giemsa and photographed. Images are representative of four independent experiments. The number of colonies was counted and the effects of the various constructs on clonogenic proliferation of MCF7 cells were analyzed as indicated elsewhere and reported in Table 1. (c) MCF10A cells were infected with recombinant retroviruses based on the pLXSN vector as indicated. Infected cells were selected using G418 and colonies of infected, drug-resistant cells were stained using Giemsa and photographed. Images are representative of at least three independent experiments. The number of colonies was counted and the effects of the various constructs on clonogenic proliferation of MCF10A cells were analyzed as indicated elsewhere and reported in Table 2.

In agreement with these published results, here we show that, relative to control constructs, the ErbB4 Q646C mutant inhibits clonogenic proliferation of (or displays tumor suppressor activity in) MCF7 human breast tumor cells (Figure 2a and 2b, Table 1) and of MCF10A human breast epithelial cells (Figure 2c, Table 2). Also consistent with published data [42, 44], the K751M mutation, which disrupts tyrosine kinase activity, disrupts the tumor suppressor activity of the ErbB4 Q646C mutant in the MCF7 and MCF10A cells (Figure 2, Table 1 and Table 2). The V673I, LL783/4AA, and L985A mutations presumably abrogate ErbB4 cleavage by gamma-secretase [10], prevent ErbB4 interactions with steroid hormone receptors [13], and prevent ErbB4 interactions with Bcl family proteins [53], respectively. These mutations disrupt inhibition of clonogenic proliferation by (disrupt tumor suppressor activity of) the ErbB4 Q646C mutant (Figure 2, Table 1, and Table 2), thereby suggesting the importance of these putative motifs for the tumor suppressor activity of the constitutively-active ErbB4 Q646C mutant.

### The V673I, LL783/4AA, and L985A mutations do not markedly alter the expression or tyrosine phosphorylation of the constitutively-active ErbB4-Q646C construct

**Figure 3:**
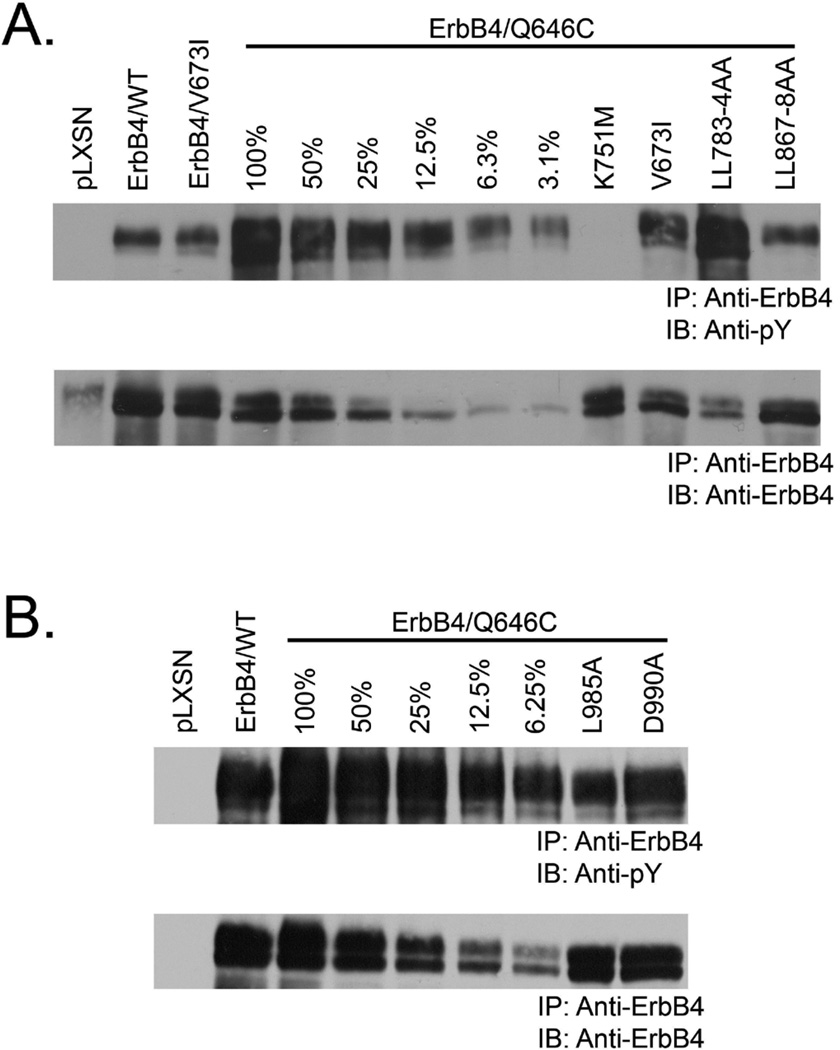
The V673I, LL783/4AA, and L985A mutations do not markedly alter the expression or tyrosine phosphorylation of the constitutively-active ErbB4 Q646C construct The expression and tyrosine phosphorylation of ErbB4 mutants expressed in Ψ^2^ cell lines generated in the course of the experiments described in Figure 2 were analyzed by ErbB4 immunoprecipitation of Ψ^2^ cell lysates and ErbB4 or anti-phosphotyrosine immunoblotting as described previously [40, 41]. These experiments were performed using 1 mg of lysate unless otherwise noted (100% indicates that 1 mg of lysate was used, 50% indicates that 0.5 mg of lysate was used, 25% indicates that 0.25 mg of lysate was used, and so on). Images are representative of three independent experiments.

Another explanation for the potential disruption of ErbB4 tumor suppressor activity by the V673I, LL783/4AA, and L985A mutations is that these mutations may abrogate ErbB4 expression and/or tyrosine phosphorylation. However, the V673I (Figure 3a), LL783/4AA (Figure 3a), and L985A (Figure 3b) mutations do not disrupt the expression or tyrosine phosphorylation of the constitutively-active ErbB4 Q646C mutant. Therefore, the abrogation of tumor suppressor activity of the constitutively-active ErbB4 Q646C mutant by the V673I, LL783/4AA, and L985A mutants appears to be due to the disruption of the gamma-secretase cleavage site, the amino-terminal steroid hormone binding motif, and the BH3 motif, respectively.

### A C-terminal EGFP tag abrogates the tumor suppressor activity of the ErbB4 Q646C mutant, but is rescued by adding a TVV tag

**Figure 4:**
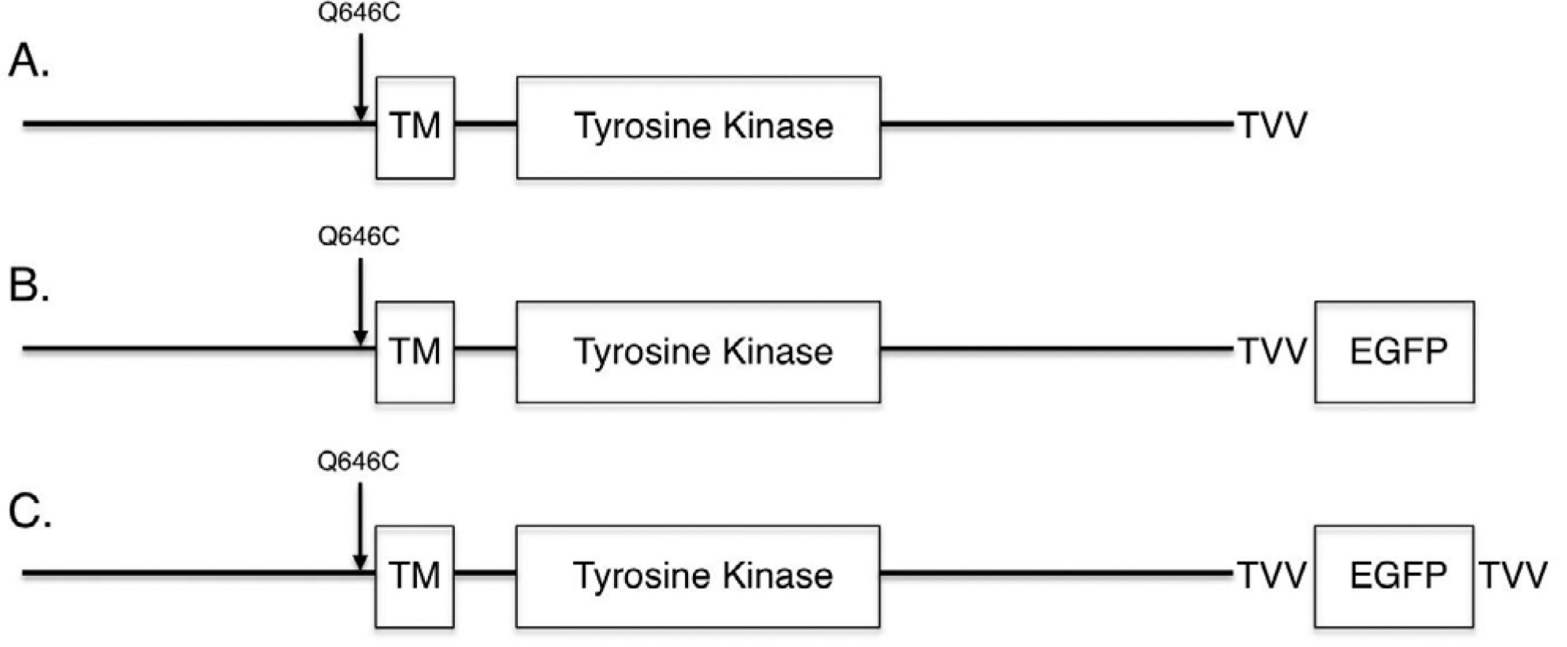
An Enhanced Green Fluorescent Protein (EGFP) tag and the amino acid sequence Thr-Val-Val (TVV) have been added to the carboxyl terminus of the ErbB4 Q646C mutant Standard subcloning techniques were used to move the EGFP sequence from the ErbB4-EGFP construct [10] to the ErbB4 Q646C construct [41], thereby generating the ErbB4 Q646C EGFP construct. Standard site-directed mutagenesis techniques were used to add the Thr-Val-Val (TVV) sequence to the extreme carboxyl terminus of the ErbB4 Q646C EGFP construct, thereby generating the ErbB4 Q646C EGFP-TVV construct.

**Figure 5:**
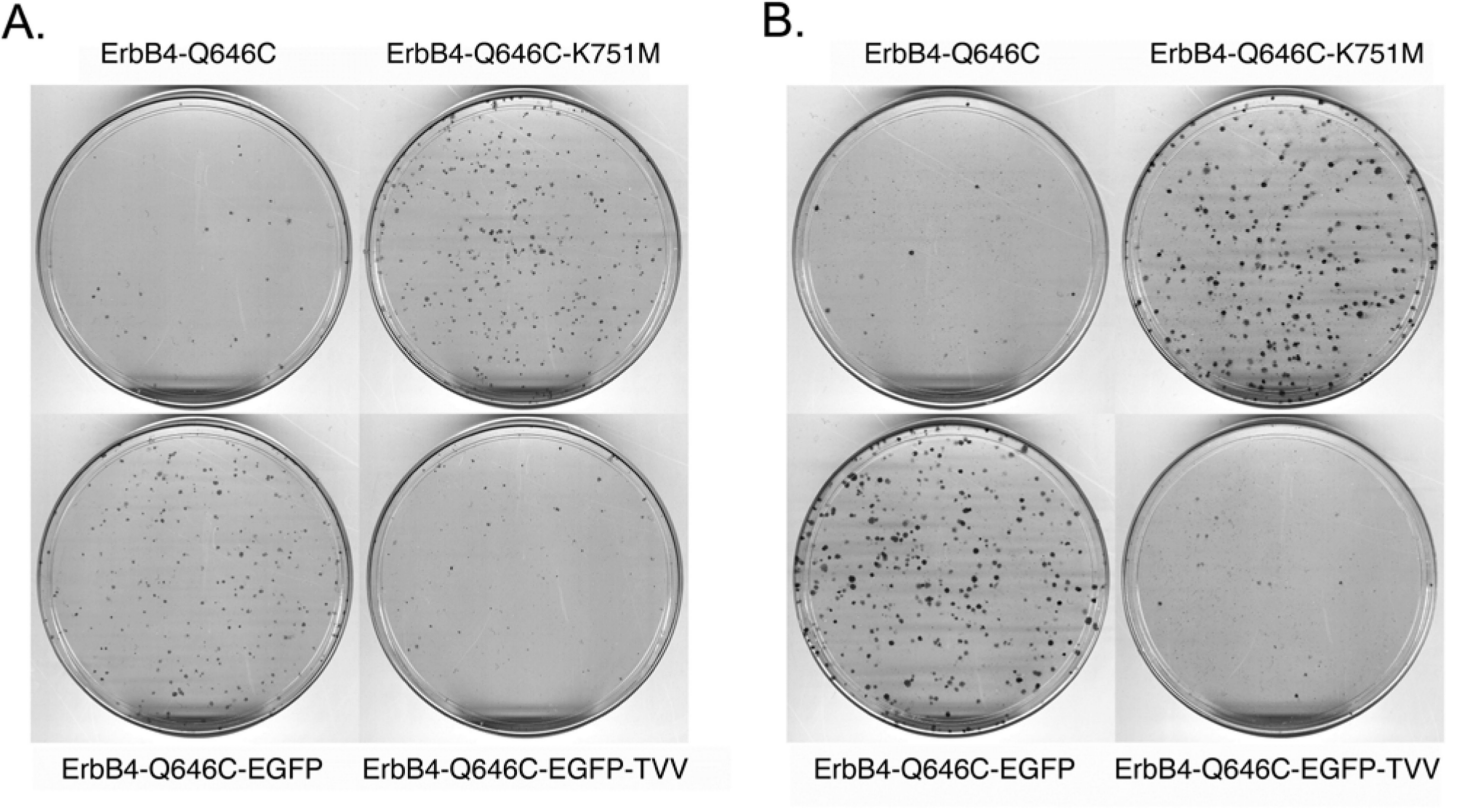
Adding a carboxyl-terminal Enhanced Green Fluorescent Protein (EGFP) tag to the constitutively-active ErbB4 Q646C mutant disrupts its tumor suppressor activity, but this deficit is rescued by adding a carboxyl-terminal Thr-Val-Val (TVV) sequence (a) PC-3 cells were infected with recombinant retroviruses based on the pLXSN-ErbB4 vector as indicated. Infected cells were selected using G418 and colonies of infected, drug-resistant cells were stained using Giemsa and photographed. Images are representative of three independent experiments. The number of colonies was counted and the effects of the various constructs on clonogenic proliferation of PC-3 cells were analyzed as indicated elsewhere and reported in Table 3. (b) DU-145 cells were infected with recombinant retroviruses based on the pLXSN vector as indicated. Infected cells were selected using G418 and colonies of infected, drug-resistant cells were stained using Giemsa and photographed. Images are representative of four independent experiments. The colonies were counted and the effects of the various constructs on clonogenic proliferation of DU-145 cells were analyzed as indicated elsewhere and reported in Table 3.

The data shown thus far suggests that the tumor suppressor activity of the constitutively-active ErbB4 Q646C mutant is dependent on cleavage by gamma-secretase and on interactions of ErbB4 with steroid hormone receptors and Bcl family proteins. Thus, we postulated that the tumor suppressor activity of the ErbB4 Q646C mutant might be dependent on subcellular trafficking of ErbB4 away from the plasma membrane and to the cytoplasm, the nucleus, and the mitochondria. To test this hypothesis, we introduced the Q646C mutation into an ErbB4 construct that possesses an Enhanced Green Fluorescent Protein (EGFP) tag at the carboxyl-terminus of ErbB4 (Figure 4). Unfortunately, unlike the parental ErbB4 Q646C construct, the resulting ErbB4 Q646C EGFP construct fails to effectively inhibit clonogenic proliferation of PC-3 (Figure 5a) or DU-145 (Figure 5b) human prostate tumor cell lines (Table 3). The carboxyl-terminal Thr-Val-Val (TVV) motif of ErbB4 regulates ErbB4 trafficking, presumably by interacting with proteins that possess PDZ domains [17, 18]. Therefore we postulated that adding a TVV sequence to the carboxyl terminus of the ErbB4 Q646C EGFP construct would rescue its tumor suppressor activity. Indeed, the ErbB4 Q646C EGFP-TVV construct inhibited clonogenic proliferation of PC-3 (Figure 5a) or DU-145 (Figure 5b) human prostate tumor cell lines to almost the same extent as the untagged ErbB4 Q646C construct (Table 3). Therefore, the ErbB4 Q646C EGFP-TVV construct is a tool that can be used to assess the effects of ErbB4 functional motif mutations on the subcellular localization and tumor suppressor activity of the ErbB4 Q646C mutant.

### The K751M, V673I, LL783/4AA, L985A, and Y1056F mutations disrupt the tumor suppressor activity of the ErbB4 Q646C EGFP-TVV construct

**Figure 6:**
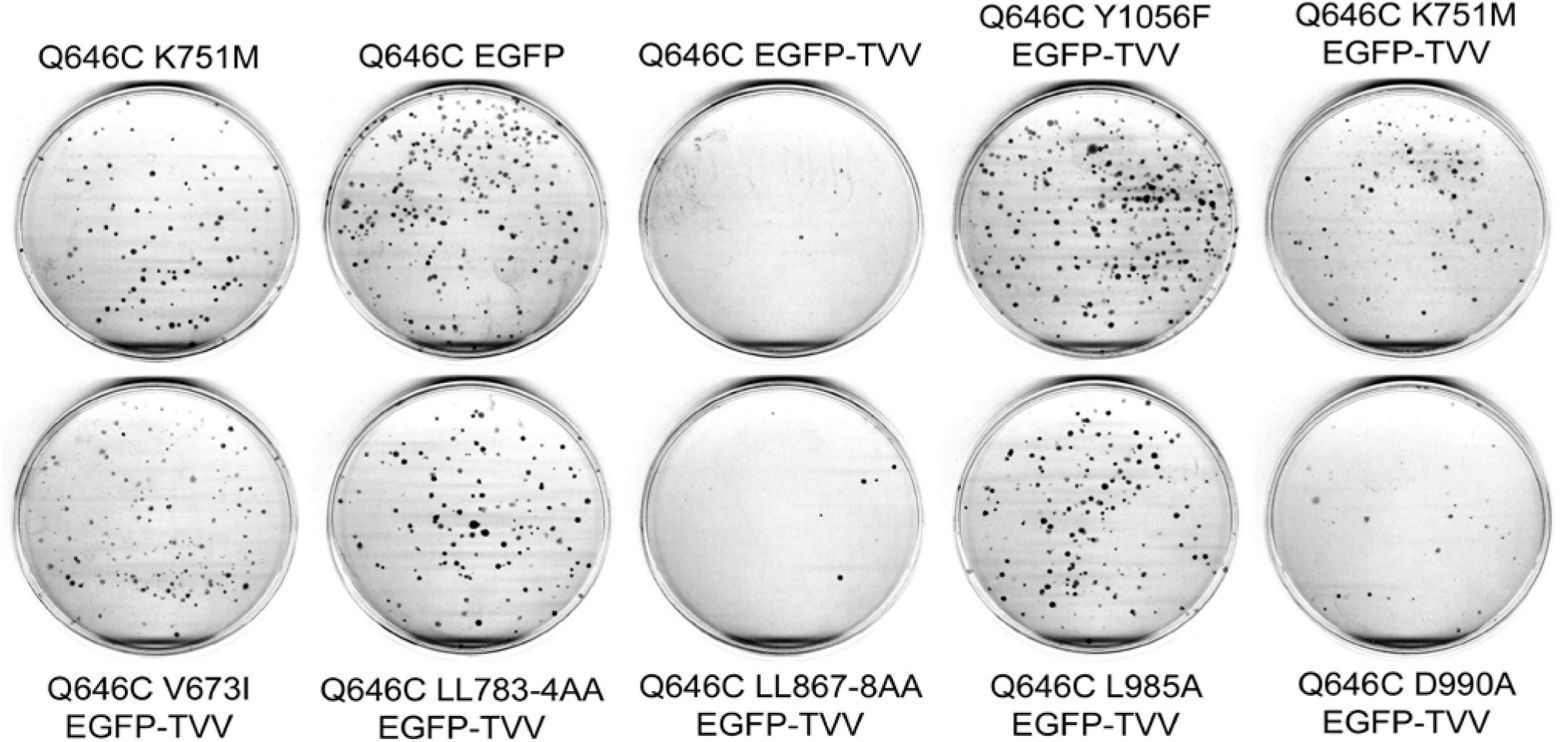
The K751M, V673I, LL783/4AA, and Y1056F mutations markedly disrupt the tumor suppressor activity of the constitutively-active ErbB4 Q646C EGFP-TVV construct in the PC-3 human prostate tumor cell line PC-3 cells were infected with recombinant retroviruses based on the pLXSN-ErbB4 vector as indicated. Infected cells were selected using G418 and colonies of infected, drug-resistant cells were stained using Giemsa and photographed. Images are representative of five independent experiments. The number of colonies was counted and the effects of the various constructs on clonogenic proliferation of PC-3 cells were analyzed as indicated elsewhere and reported in Table 4.

**Figure 7:**
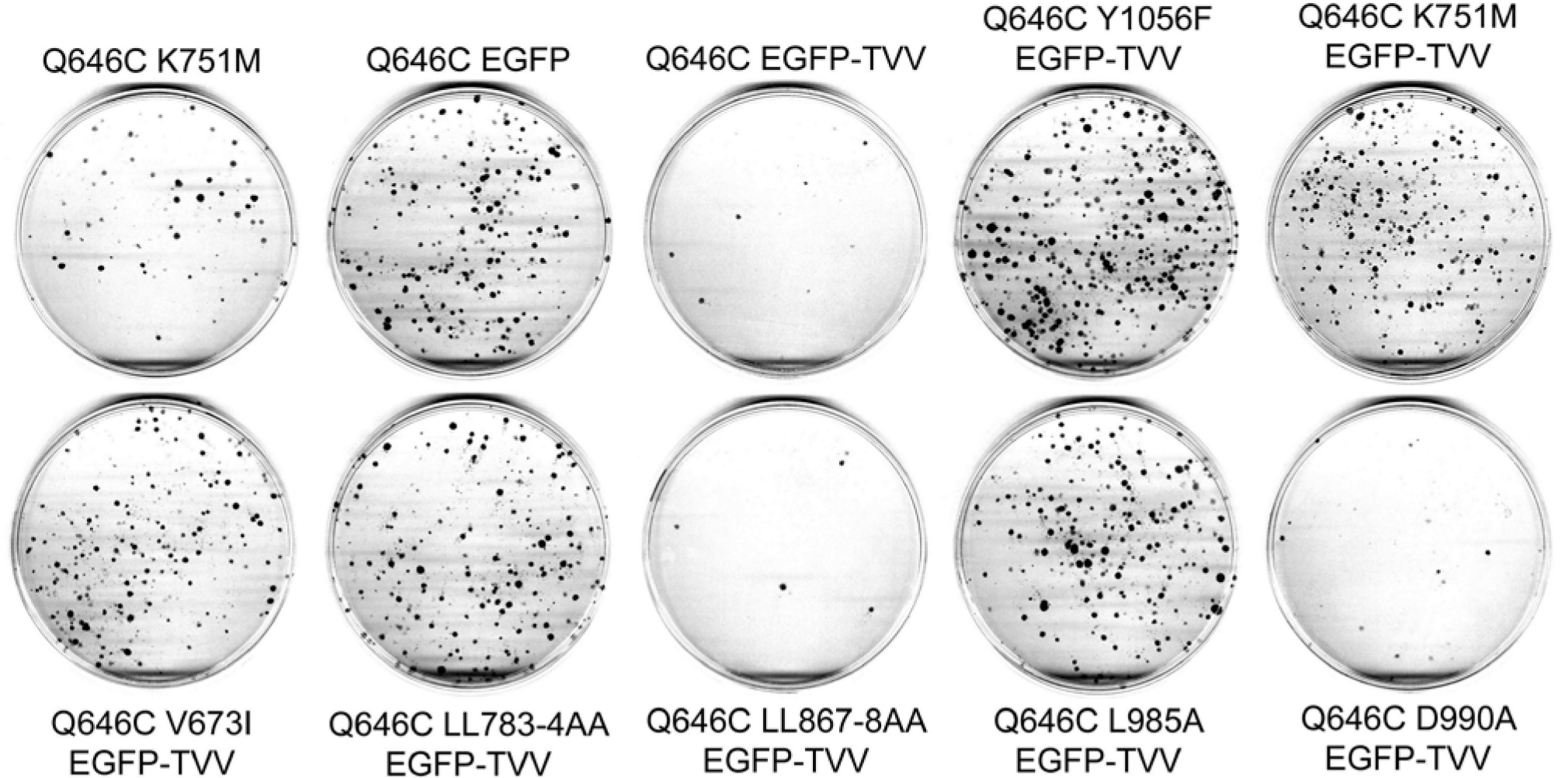
The K751M, V673I, LL783/4AA, and Y1056F mutations markedly disrupt the tumor suppressor activity of the constitutively-active ErbB4 Q646C EGFP-TVV construct in the DU-145 human prostate tumor cell line DU-145 cells were infected with recombinant retroviruses based on the pLXSN vector as indicated. Infected cells were selected using G418 and colonies of infected, drug-resistant cells were stained using Giemsa and photographed. Images are representative of five independent experiments. The colonies were counted and the effects of the various constructs on clonogenic proliferation of DU-145 cells were analyzed as indicated elsewhere and reported in Table 4.

In results that are similar to those shown elsewhere in this report, mutations that disrupt ErbB4 kinase activity (K751M), ErbB4 cleavage by gamma-secretase (V673I), ErbB4 interactions with steroid hormone receptors (LL783/4AA), ErbB4 interactions with Bcl family proteins (L985A), and ErbB4 phosphorylation at Tyr1056 (Y1056F) all disrupt inhibition of clonogenic proliferation of (or disrupt tumor suppressor activity in) the PC-3 (Figure 6) and DU-145 (Figure 7) human prostate tumor cell lines (Table 4). Thus, ErbB4 kinase activity, ErbB4 cleavage by gamma-secretase, ErbB4 interactions with steroid hormone receptors, ErbB4 interactions with Bcl family proteins, and interactions with effectors via binding to ErbB4 phospho-Tyr1056 all appear to be critical for the tumor suppressor activity of the constitutively-active ErbB4 Q646C EGFP-TVV construct.

### The K751M, V673I, and Y1056F mutations disrupt the subcellular localization of the ErbB4 Q646C EGFP-TVV construct

**Figure 8:**
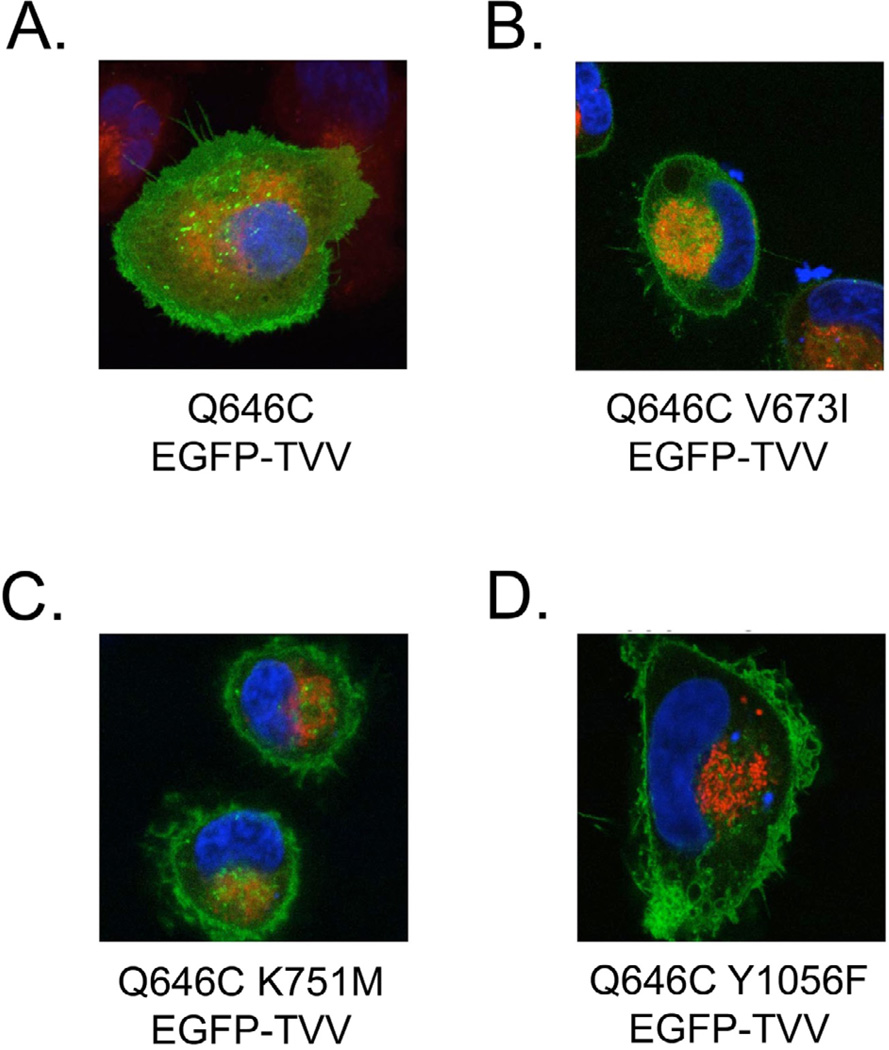
The K751M, V673I, and Y1056F mutations disrupt the subcellular localization of the ErbB4 Q646C EGFP-TVV construct The various ErbB4 Q646C EGFP-TVV constructs were transiently transfected into the PC-3 human prostate tumor cell line, after which the cells were stained with Hoescht 33342 (for DNA - blue) or MitoTracker Red CMXRos (for mitochondria - red). Cells were imaged by laser scanning confocal microscopy; the EGFPtagged proteins appear as green in these images. We photographed multiple randomly-selected, EGFP-positive cells per transfected plasmid per experiment. Images are representative of at least three independent experiments.

Next, we used confocal microscopy to assess whether ErbB4 mutations that disrupt the tumor suppressor activity of the constitutively-active ErbB4 Q646C EGFP-TVV construct also alter its subcellular localization (Figure 8). Imaging of the EGFP tag reveals that the positive control ErbB4 Q646C EGFP-TVV construct displays plasma membrane expression as well as both diffuse and punctate expression throughout the cytoplasm (Figure 8), suggesting that ErbB4 is cleaved and trafficked away from the plasma membrane. In contrast, the K751M, V673I, and Y1056F mutants predominantly exhibit plasma membrane expression, only modest diffuse cytoplasmic expression, and little punctate cytoplasmic expression (Figure 8). This suggests that the absence of tumor suppressor activity displayed by these mutants may be a result of ErbB4 mislocalization. The L985A and LL783/4AA mutations, which disrupt the tumor suppressor activity of the ErbB4 Q646C EGFP-TVV construct, retain cytoplasmic expression (data not shown), suggesting that ErbB4 trafficking to the cytoplasm is necessary, but not sufficient, for the tumor suppressor activity of the ErbB4 Q646C EGFP-TVV construct. Inadequate expression or resolution or repression of an active translational event may account for the failure to observe nuclear or mitochondrial expression of the ErbB4 Q646C EGFP-TVV construct. However, we note that disrupting the BH3 domain or the amino-terminal LXXLL motif of ErbB4 abrogates the tumor suppressor activity of the ErbB4 Q646C mutant, suggesting that ErbB4 targeting to the nucleus and mitochondria is important for the tumor suppressor activity of the ErbB4 Q646C mutant. Because the ErbB4 Q646C EGFP-TVV construct retains tumor suppressor activity, it is reasonable to postulate that it too undergoes translocation to the nucleus and mitochondria and that inadequate expression or resolution accounts for the failure to observe this translocation.

## Discussion

### Multiple functional motifs are required for the tumor suppressor activity of the constitutively-active ErbB4 Q646C mutant

Protein sequence and functional analyses suggest that ErbB4 possesses multiple functional motifs that are responsible for coupling ErbB4 to signaling effectors and biological responses (Figure 1). Here we have used mutations that target these putative functional motifs to demonstrate that several of these putative motifs are required for the tumor suppressor activity of the constitutively-active ErbB4 Q646C mutant.

These results suggest that ErbB4 kinase activity and ErbB4 cleavage by gamma-secretase are both required for the tumor suppressor activity of the constitutively-active ErbB4 Q646C mutant. Likewise, ErbB4 interactions with a steroid hormone receptor(s) and with a Bcl family protein(s) also appear to be required for the tumor suppressor activity of the constitutively-active ErbB4 Q646C mutant. (These interactions appear to be site specific, as the LL867/8AA and D990A mutants retain at least some tumor suppressor activity.) Finally, the interaction between the ErbB4 phosphorylation site at Tyr1056 and an effector protein(s) appears to be required for the tumor suppressor activity of the constitutively-active ErbB4 Q646C mutant.

### Alterations in ErbB4 subcellular localization may contribute to changes in ErbB4 function

Localization data presented here suggest that the ErbB4 motifs required for the tumor suppressor activity of the constitutively-active ErbB4 Q646C mutant may couple to this tumor suppressor activity in part by regulating ErbB4 cleavage and intracellular trafficking. This allows us to postulate a model that integrates ErbB4 trafficking, ErbB4 binding to effector proteins, and ErbB4 signaling activity. For example, the V673I mutation is expected to block ErbB4 cleavage by gamma-secretase, thereby preventing trafficking of the ErbB4 cytoplasmic domains away from the plasma membrane and to the cytoplasm, mitochondria, or nucleus. Similarly, the LL783/4AA mutation is expected to block interactions between ErbB4 and steroid hormone receptors, and thereby prevent steroid hormone receptors from directing ErbB4 to the nucleus. Finally, the L985A mutation is expected to block interactions between ErbB4 and Bcl family proteins, and thereby prevent Bcl family proteins from directing ErbB4 to the mitochondria.

It is not surprising that targeting the canonical site of ErbB4 binding (at ErbB4 phospho-Tyr1056) to the regulatory subunit of the PI3 kinase (through either the Y1056F or K751M mutations) disrupts the tumor suppressor activity of the constitutively-active ErbB4 Q646C mutant; the Y1056F or K751M mutations are predicted to prevent ErbB4 from coupling to PI3 kinase activity and to its pro-proliferative, anti-apoptotic effectors. However, it is somewhat surprising that the Y1056F mutant and the K751M mutant both fail to display cytoplasmic localization. This suggests that ErbB4 phosphorylation at Tyr1056 and consequent binding to the regulatory subunit of PI3 kinase is required for ErbB4 cleavage and/or trafficking away from the plasma membrane. This is somewhat at odds with the evidence suggesting that PI3 kinase functions primarily at the plasma membrane [58]. Indeed, another possibility may be that preventing phosphorylation at Tyr1056 of ErbB4 alters ErbB4 binding to effectors that possess a WW domain(s) and that this change in effector binding alters the cleavage and/or intracellular trafficking of ErbB4.

### Heterodimerization of ErbB2 with ErbB4 may alter ErbB4 function by changing ErbB4 trafficking

We have previously demonstrated that in human pancreatic tumor cell lines in which the constitutively-active ErbB4 Q646C mutant functions as a tumor suppressor, wild-type ErbB4 appears to possess oncogenic activities [59]. We have attempted to rationalize this apparent paradox by demonstrating that the ErbB4 Q646C mutant functions as a homodimer in these cells, whereas wild-type ErbB4 functions as a heterodimer with EGFR or ErbB2. Specifically, we demonstrated that the oncogenic activities of wild-type ErbB4 require ErbB4 sites of tyrosine phosphorylation and EGFR or ErbB2 kinase activity, but not ErbB4 kinase activity. We postulated that EGFR or ErbB2 phosphorylates a set of ErbB4 tyrosine residues that is distinct from the set that are phosphorylated by ErbB4 itself [59]. Given the ErbB4 localization data shown in the work at hand, we now postulate that ErbB4 trafficking following heterodimerization with EGFR or ErbB2 may be quite different from ErbB4 trafficking resulting from ErbB4 homodimerization. Furthermore, this difference in trafficking may account for the functional differences between ErbB4 homodimers and ErbB2/ErbB4 or EGFR/ErbB4 heterodimers. Consequently, additional experimentation is warranted to further elucidate the roles that intracellular trafficking plays in regulating ErbB4 function, particularly in the context of ErbB4 homodimers and heterodimers.

## Figures and Tables

**Table 1:** The K751M, V673I, LL783/4AA, and L985A mutations markedly disrupt the tumor suppressor activity of the constitutively-active ErbB4 Q646C mutant in the MCF7 human breast tumor cell line.

Cell Line	Retrovirus	Clonogenic Efficiency (Relative to C127 Cells)	Inhibition of Clonogenicity (Relative to LXSN)
MCF7 (n=4)	LXSN	2.0%	N/A
	ErbB4	1.6%	15 ± 2%
	ErbB4 Q646C	0.04%	97 ± 1%
	ErbB4 Q646C K751M	3.3%	None
	ErbB4 Q646C V673I	2.5%	None
	ErbB4 Q646C LL783/4AA	2.4%	None
	ErbB4 Q646C LL867/8AA	0.5%	70 ± 7%
	ErbB4 Q646C L985A	2.9%	None
	ErbB4 Q646C	1.2%	35 ± 13%

**Table 2:** The K751M, V673I, LL783/4AA, and L985A mutations markedly disrupt the tumor suppressor activity of the constitutively-active ErbB4 Q646C mutant in the MCF10A human breast epithelial cell line.

Cell Line	Retrovirus	Clonogenic Efficiency (Relative to C127 Cells)	Inhibition of Clonogenicity (Relative to LXSN)
Expt. 1 MCF10A (n=4)	LXSN	9.8%	N/A
	ErbB4	10.0%	None
	ErbB4 Q646C	0.8%	91 ± 1%
	ErbB4 Q646C K751M	8.5%	None
	ErbB4 Q646C V673I	8.5%	None
Expt. 2 MCF10A (n=3)	LXSN	4.3%	N/A
	ErbB4	6.2%	None
	ErbB4 Q646C	0.5%	87 ± 2%
	ErbB4 Q646C K751M	9.5%	None
	ErbB4 Q646C LL783/4AA	7.4%	None
	ErbB4 Q646C LL867/8AA	1.8%	58 ± 6%
Expt. 3 MCF10A (n=3)	LXSN	5.8%	N/A
	ErbB4	7.1%	None
	ErbB4 Q646C	0.6%	88 ± 3%
	ErbB4 Q646C K751M	7.4%	None
	ErbB4 Q646C L985A	5.8%	None
	ErbB4 Q646C D990A	4.4%	28 ± 13%

**Table 3:** Adding a carboxyl-terminal Enhanced Green Fluorescent Protein (EGFP) tag to the constitutively-active ErbB4 Q646C mutant disrupts its tumor suppressor activity, but this deficit is rescued by adding a carboxylterminal Thr-Val-Val (TVV) sequence.

Cell Line	Retrovirus	Clonogenic Efficiency (Relative to C127 Cells)	Inhibition of Clonogenic Proliferation (Relative to ErbB4 Q646C K751M)
DU-145 (n=4)	ErbB4 Q646C 751M	7.4%	N/A
	ErbB4 Q646C	0.3%	95 ± 2%
	ErbB4 Q646C EGFP	6.0%	15 ± 9%
	ErbB4 Q646C EGFP-TVV	1.2%	83 ± 4%
PC-3 (n=3)	ErbB4 Q646C K751M	12.0%	N/A
	ErbB4 Q646C	1.4%	90 ± 3%
	ErbB4 Q646C EGFP	10.9%	15 ± 8%
	ErbB4 Q646C EGFP-TVV	1.1%	89 ± 3%

**Table 4:** The K751M, V673I, LL783/4AA, and Y1056F mutations markedly disrupt the tumor suppressor activity of the constitutively-active ErbB4 Q646C EGFP-TVV construct in the DU-145 and PC-3 human prostate tumor cell lines.

Cell Line	Retrovirus	Clonogenic Efficiency (Relative to C127 Cells)	Inhibition of Clonogenic Proliferation (Relative to ErbB4 Q646C K751M)
DU-145 (n=5)	ErbB4 Q646C K751M	4.6%	N/A
	ErbB4 Q646C EGFP	5.5%	None
	ErbB4 Q646C EGFP-TVV	0.8%	82 ± 2%
	ErbB4 Q646C K751M EGFP-TVV	12.8%	None
	ErbB4 Q646C V673I EGFP-TVV	5.2%	None
	ErbB4 Q646C LL783/4AA EGFP-TVV	8.9%	None
	ErbB4 Q646C LL867/8AA EGFP-TVV	0.4%	92 ± 2%
	ErbB4 Q646C L985A EGFP-TVV	2.8%	35 ± 10%
	ErbB4 Q646C D990A EGFP-TVV	1.4%	69 ± 4%
	ErbB4 Q646C Y1056F EGFP-TVV	9.7%	None
PC-3 (n=5)	ErbB4 Q646C K751M	15.7%	N/A
	ErbB4 Q646C EGFP	24.9%	None
	ErbB4 Q646C EGFP-TVV	2.0%	89 ± 3%
	ErbB4 Q646C K751M EGFP-TVV	22.9%	None
	ErbB4 Q646C V673I EGFP-TVV	8.9%	28 ± 17%
	ErbB4 Q646C LL783/4AA EGFP-TVV	26.1%	None
	ErbB4 Q646C LL867/8AA EGFP-TVV	3.8%	80 ± 3%
	ErbB4 Q646C L985A EGFP-TVV	8.5%	49 ± 6%
	ErbB4 Q646C D990A EGFP-TVV	3.3%	78 ± 3%
	ErbB4 Q646C Y1056F EGFP-TVV	23.4%	None
